# Sex differences in adolescents’ occupational aspirations: Variations across time and place

**DOI:** 10.1371/journal.pone.0261438

**Published:** 2022-01-26

**Authors:** Gijsbert Stoet, David C. Geary

**Affiliations:** 1 Department of Psychology, University of Essex, Colchester, United Kingdom; 2 Department of Psychological Sciences, University of Missouri-Columbia, Columbia, Missouri, United States of America; University of Gothenburg: Goteborgs Universitet, SWEDEN

## Abstract

We investigated sex differences in 473,260 adolescents’ aspirations to work in things-oriented (e.g., mechanic), people-oriented (e.g., nurse), and STEM (e.g., mathematician) careers across 80 countries and economic regions using the 2018 Programme for International Student Assessment (PISA). We analyzed student career aspirations in combination with student achievement in mathematics, reading, and science, as well as parental occupations and family wealth. In each country and region, more boys than girls aspired to a things-oriented or STEM occupation and more girls than boys to a people-oriented occupation. These sex differences were larger in countries with a higher level of women’s empowerment. We explain this counter-intuitive finding through the indirect effect of wealth. Women’s empowerment is associated with relatively high levels of national wealth and this wealth allows more students to aspire to occupations they are intrinsically interested in. Implications for better understanding the sources of sex differences in career aspirations and associated policy are discussed.

## Introduction

The psychological traits that influence people’s occupational aspirations are of substantive theoretical and practical importance. These traits influence a major aspect of one’s long-term economic prospects in life, sit at the juncture of research between differential psychology and labor economics, and often have important policy implications. One associated and often contentious question concerns the sex difference in occupational interests, which is possibly "the largest of all sex differences on major psychological dimensions" [1]. These sex differences are well established; they have been studied for more than a century and are relatively consistent across nations and across historical periods [2–5]. The sex differences question is often contentious because men are overrepresented in many high-paying and high-status science, technology, engineering, and mathematics (STEM) occupations despite increased legal, political, and socioeconomic gender equality [6]. This runs counter to the assumption that increased levels of gender equality in political, economic, and educational participation would lead to greater similarities in women’s and men’s psychological traits and, thus, reduce gender stratification in occupational choices [7]. Thus, a more complete understanding of sex differences in occupational preferences provides insights into the factors that influence the expression of sex differences and could be useful for some people attempting to manage gender disparities in occupational paths.

Here, we use the latest data from the Programme for International Student Assessment (PISA [8]) to characterize the cross-national pattern of sex differences in adolescents’ occupational aspirations and to test related hypotheses derived from relevant psychological theories. The triennial PISA survey compares the academic achievement and related traits (e.g., interests) of students in the world’s most economically developed nations. One goal of the sponsoring Organisation for Economic Collaboration and Development (OECD) is to set social benchmarks, including fostering equal educational and later occupational opportunities for girls and boys [9]. In keeping with this goal, we integrated the assessment of occupational aspirations with sex differences in academic strengths, parental socioeconomic status, and country-level factors (e.g., women’s political and economic opportunities) to provide a thorough and unique analysis of sex differences in the factors that presage later occupational segregation.

### Sex differences in occupational interests and choices

Sex differences in occupational (a.k.a. vocational) interests have been studied for well over a century. For example, King (1914 [2]) surveyed 200 high school students’ vocational and school-subject interests and found that girls, on the whole, expressed little interest in becoming an engineer or mechanic. In a 1918 assessment of 1,666 adolescents, Miner [10] found large sex differences in occupational aspirations, including 11.55 boys to every girl preferring to work with engines and 12.46 girls to every boy preferring to work in teaching. Overall, working with people was preferred 3.7 to 1 over working with things for girls but boys, as a group, had similar interests in people-oriented as contrasted with things-oriented work. Sex differences were even evident within more people-oriented jobs, with girls focused more on jobs that involved direct interpersonal engagement with others (e.g., teaching, working in welfare organizations) and boys on higher-status (e.g., physician) or entrepreneurial (e.g., sales) jobs. Carter and Strong [11] found the same in a 1933 study, namely that adolescent girls, overall, reported a greater interest in working with people than did boys.

These studies were possibly the first explicit referrals to a people-things dimension contributing to the sex differences in occupational interests. These sex differences were further confirmed by Finch and Odoroff in 1939 [12] or more recently by Mozahem and colleagues in Lebanon (*d* = 0.8) [13]. On the basis of these findings it is not surprising that today, many of the occupational sex differences (e.g., few female electricians) have been explained by boys’ and men’s greater interest in things (i.e., mechanical tools, machines, or gadgets) as opposed to an interest in helping people or living beings more broadly, and the reverse for girls and women [3–5, 14, 15].

Despite the remarkable stability over time of the sex differences in occupational interests, there have been important secular changes in girls’ and women’s broad vocational preferences. In recent decades, girls and women have expressed more interest in the people-oriented occupations of medicine and veterinary science than have men, whereas none of the adolescent girls in Winston’s 1935 [16] and King’s 1914 study [2] chose medicine. This demonstrates that at least some aspects of people’s vocational interests change over generations.

The results from these early studies were confirmed in Su and colleagues’ [4] large meta-analytical study, spanning four decades of research; there are large sex differences in vocational interests in people-oriented and things-oriented occupations across nations (*d* = 0.93). In keeping with these differences, Lippa, Preston, and Penner’s [3] study of the US labour market from 1972 to 2010 showed a secular increase in women’s representation in higher-status jobs (e.g., lawyer) but no change in women’s representation in things-oriented jobs (e.g., automotive mechanics). Across cultures, however, there is considerable variation in women’s participation in things-oriented jobs, with higher participation in less economically developed nations [3]. Lippa and colleagues suggested that the greater relative employment of women in things-oriented jobs in less developed nations was a matter of economic necessity rather than of interest (see also [6]). In other words, secular improvements in general economic development and in women’s occupational opportunities resulted in their movement into higher-status but not things-oriented occupations (in the 1972 to 2010 period).

Despite the long-term and widespread study of the sex differences in interest in people-oriented as compared to things-oriented occupations, there is no consensus on the sources of these differences. The associated studies range from a focus on stereotypes (e.g., [17]) to prenatal exposure to androgens (e.g., [18]) and, as noted, are often controversial (for an overview, see [19]).

### Terminology

Before turning to the details of the current project, we note that the study of sex differences in occupational choice is complicated by ambiguity in the associated terms. In today’s educational policy discussions and academic research about sex differences and education, the term STEM is often used, even though this covers only a small section of the labor force (e.g., [20–22]).

Despite the common usage of the term STEM, it is not well-defined. It has been used at an institutional level at least since 1992 by the US-based Center for the Advancement of Hispanics in Science and Engineering Education and referred exclusively to non-biological science, mathematics and technology [23, 24]. Since then, there have been debates about the STEM inclusion of social sciences and psychology [25] and whether certain health professions (i.e., doctors, nurses, and veterinarians) should be included, as suggested by the 2018 PISA documentation [26].

The attempts to broaden STEM do not include many less prestigious, yet vital, blue-collar technical occupations in which women are strongly underrepresented. Examples are car repair, welding, plumbing, and electrical work. One result is that STEM fields are not fully representative of the general population; discussions around STEM participation focuses on white-collar occupations more frequently aspired to by students from higher socioeconomic groups. For example, in the 2018 PISA data, students choosing blue-collar technical (things-oriented) occupations not counted as STEM (e.g., welder and flame cutter) are from a considerably lower socioeconomic family background than their peers choosing white-collar STEM occupations (e.g., engineer).

To address these limitations in the definition of STEM, we argue that studying broader and more established categories, namely things-oriented and people-oriented careers will provide a more complete understanding of the sex differences in occupational aspirations and the resulting knowledge/skills gaps. Not only do the categories "things-oriented occupation" and "people-oriented occupation" build on a longer tradition of research [3–5, 14, 15], they also are more inclusive of the entire socioeconomic distribution of occupations. This means that academics and policymakers with an interest in understanding sex-specific interests and knowledge/skills gaps benefit from a broader spectrum of occupations than would be possible with a STEM-only focus.

### The current study

Focusing on broad technical occupations (in addition to the narrow original STEM range) integrates the present study with the extensive research on sex differences in the people-things dimension of occupational preferences [4, 27], and at the same time adds substantively to this literature. The large, representative and cross-national samples enabled us to examine sex difference and individual-level (e.g., academic strengths, parental occupation) correlates of adolescents’ occupational aspirations, as well as national-level moderators (e.g., degree of women’s empowerment) of the strength of the sex differences in these aspirations.

As noted, the 2018 PISA asked adolescents to report the occupations they expected to work in when they were about 30 years old. We classified these occupations as being "things oriented", "people oriented", or "other" (see Methods), as well as being STEM or not. Things-oriented occupations are those that involve extensive work with machines, such as computer programming, repairing machines (e.g., cars), or tailoring, whereas people-oriented occupations involve beneficial face-to-face interactions, as in medicine or teaching. Many occupations cannot clearly be classified as one or the other, such as restaurant managers. These positions have a social component to them, but also important non-social components (e.g., managing stock, schedules, payroll). Ultimately, the people vs things dimension is a continuous scale, but in our analyses we only used categories that are predominantly one or the other. All things-oriented jobs have a clear technical component, ranging from locomotive engine driver to astronomer and all people-oriented jobs have a clear component of providing help to individuals.

We used the PISA data to test several hypotheses regarding sex differences in preferences for things-oriented, people-oriented, and STEM occupations.

First, we hypothesized that the previously reported sex differences in the people-things occupational preferences as well as STEM preferences are also reflected in adolescents’ occupational aspirations and are found across nations. This hypothesis assumes that sex differences in occupational aspirations are not only the result of barriers experienced by those entering or already in the labor market, but also by sex differences in the psychological traits that make some people more attracted to certain occupations than others [28].

Second, we hypothesized that the magnitude of the sex differences in occupational aspirations varies systematically between nations. More specifically, we hypothesized that the sex differences in these preferences are larger in countries with greater levels of women’s empowerment, as differences in many domains tend to be larger in these contexts [3, 6, 29, 30]. This effect was named the "gender equality paradox" by Stoet and Geary [6], who found that the gap in STEM graduation rates (i.e., proportionately more men than women) was larger in countries with a higher degree of women’s political, economic, and educational empowerment (as expressed by the commonly used Global Gender Gap Index, see Methods).

Third, we hypothesized that the counter-intuitive relation between women’s empowerment and the increase in the magnitude of sex differences in occupational interests is indirect and can be explained by the intermediary variable of national wealth. Like Lippa and colleagues [3], we propose that national wealth plays a role, because students in wealthier countries are less likely to choose occupations based largely on concerns about long-term economic security. New in our theory is a clear model on how women’s empowerment, wealth, and educational outcomes are related. We propose that empowerment of women increases national levels of wealth; this is based on the idea that when women are better educated and when women have better opportunities to work, this leads to increased economic productivity and wealth [31, 32]. Increases in wealth, in turn, result in fewer economic constraints on occupational choices (i.e., choosing an occupation largely for economic security) and enable greater freedom to make occupational choices based on personal interests.

In order to test these hypotheses, we present a detailed analysis of the relation between occupational aspirations among 15 and 16-year old boys and girls across the OECD and a number of non-OECD nations. We will also highlight a few specific cross-national differences to provide a more detailed understanding about how countries differ in the numbers of boys and girls aspiring to things-oriented, people-oriented, and STEM occupations. In the context of sociopolitical aims to increase the numbers of girls in things-oriented or STEM occupations, we analyzed which countries succeed in these aims while also scoring relatively high on other relevant variables (overall educational achievement and gender empowerment).

## Materials and methods

Our study is based on the analysis of various publicly available data sets, described below.

*PISA*: We used the 2018 PISA dataset, which included information on randomly sampled (based on lists provided by local educational authorities, *N* = 612,004) 15- and 16-year-olds from 80 countries and regions. PISA selection of students follows a relatively complex procedure to ensure random sampling and representative data from the whole population of each participating country [8].

Students completed generic tests in the domains of mathematics, reading, and science. Each student’s mathematics, reading, and science score is represented as 10 different plausible values, which were drawn from a most likely distribution of scores estimated by an item response model [33].

A subset of participants (*N* = 473,260) answered the question "What kind of job do you expect to have when you are about 30 years old?", which we call "occupational aspiration" in this study. We analyzed data of this subset of participants. The median number of students per country who answered this question was 4,826.5. The median percentage of students across countries who answered this question was 78%. A complete list of the student distribution across countries is provided in S1 Table.

The open answers to the question about job aspiration were not published; instead, the PISA team classified these and published the processed answers in accordance with the International Standard Classification of Occupations (ISCO) [34]. Each occupation has a four-digit code of which the first digit indicates the main group (such as *managers* or *plant and machine operators*, *and assemblers*). PISA’s classification of students’ occupational aspirations used 584 different occupational codes.

The main group (i.e., first digit) can be used to classify jobs as white collar (managers, professionals, technicians and associate professionals, clerical support workers, service and sales workers) or blue collar (skilled agricultural, forestry and fishery workers, craft and related trades workers, plant and machine operators, and assemblers, and elementary occupations such as cleaners), and as lower skilled (groups 4,5,8,9) or higher skilled (1,2,3,6,7). For example, ISCO code 2634 refers to "Psychologists" (white collar, higher skilled), 7231 refers to "Motor vehicle mechanics and repairers" (blue collar, higher skilled), and 8332 "Heavy truck and lorry drivers" (blue collar, lower skilled).

We first classified the ISCO codes of each of the different occupations into one of three categories: 1) Things-oriented occupations; 2) People-oriented occupations; or 3) Neither 1 or 2. A things-oriented occupation is one that is heavily focused on using (or designing/creating) a tool or machinery. This includes categories such as welding, civil engineering, or application programmer. A people-oriented occupation is one that is centered around interacting with clients, customers, or children. This includes all teaching and instructor occupations as well as all professions in which patients are being helped (therefore, we included veterinarians). Occupations that did not fit either of these categories, such as biologist, legislator, or accountant, were classified as "other". In all, 196 occupations were classified as things oriented, 76 as people oriented, and 312 as other (Table 1).

**Table 1:** Classification of 584 occupational categories derived from the ISCO categories available in the sample. For each of the three categories (things, people, other), we further list how many occupations fall in the blue/white collar and lower/higher skilled categories. Percentages are based on row totals.

	*Total*	*Blue collar*, *lower skilled*	*Blue collar*, *higher skilled*	*White collar*, *lower skilled*	*White collar*, *higher skilled*
Things jobs	196	51 (26%)	72 (37%)	0 (0%)	73 (37%)
People jobs	76	0 (0%)	0 (0%)	24 (33%)	52 (68%)
Other jobs	312	49 (16%)	38 (12%)	72 (23%)	146 (47%)

Further, we also classified STEM occupations in the ISCO classification scheme, most of which were things-oriented occupations, but not all (namely mathematician and statistician). To do so, we identified five science occupations (ISCO 2110–2114), 11 technology codes (ISCO 2510–2514, 2519, 2520–2523, and 2529), 23 engineering codes, and three mathematics codes (ISCO 2120, 3310, 3314). As noted in the introduction, this is a conservative approach to identifying STEM occupations.

PISA also contains information about the occupation of the father and mother of a student (if available). We also categorized their occupations into the same categories (i.e., things-oriented, people-oriented, other, and STEM).

In our analysis, we calculated gender ratios for things-oriented and people-oriented career aspirations as well as for STEM occupations. We did this because economic fluctuations in demand for specific occupations are less likely to distort ratios than the absolute numbers (or percentages) of women and men working in a particular field. This is especially true for some smaller economies that might be biased toward certain types of occupations. Where appropriate, we will discuss percentage of boys and girls for specific areas. Due to low numbers of STEM choices in some countries, there is considerable variation in the confidence intervals. For this reason, we exclude Japan’s STEM sex ratio, because Japan’s confidence interval is more than three times as large as the second-largest confidence interval. Japan’s gender ratio in STEM career aspirations was much larger than the second largest ratio (Vietnam, 10.9, [6.6–15.1]) and Japan’s confidence interval was 2.7 times larger than the second largest confidence interval; therefore, we excluded this imprecise outlier data point from further STEM-specific analyses.

For some analyses, we used the definition of sex-typical and sex-atypical occupations. Sex typical are things-oriented and STEM occupations aspired to by boys and people-oriented occupations by girls. Sex atypical are things-oriented and STEM occupations aspired to by girls and people-oriented occupations by boys. Note that the percentage of students not choosing sex-typical occupations includes those who choose "other" occupations, whereas the percentage of students choosing sex-atypical occupations is much smaller and only includes things-oriented, STEM, and people-oriented occupations.

We used PISA’s wealth measure to calculate a national wealth indicator, which more directly reflects wealth for the population in question than does the national Gross Domestic Product (GDP). The PISA wealth score is based on home resources, such as the number of electronic gadgets, educational materials, and whether a student has a desk of their own to study at.

For all analyses, we used the latest version of the R software [35] and we followed the statistical guidelines provided by the PISA consortium [36].

### Ethical approval

No institutional ethical approval was necessary for carrying out this secondary data analysis of the publicly available and fully anonymized PISA datasets. Parental permission for student participation in the PISA surveys was secured by the staff coordinating PISA data collection when required by schools.

### Global gender gap index

The Global Gender Gap Index (GGGI) is an annually reported measure of women’s empowerment based on gender ratios in political representation, economic participation, duration of formal education, and various health measures (e.g., healthy life span). Each country is ranked on a continuous 0 to 1 scale, with higher scores representing stronger empowerment. We used the 2018 report to match the 2018 PISA data [37]. The countries participating in the 2018 PISA ranged from 0.590 (Saudi Arabia) to 0.858 (Iceland).

### Global innovation index

The Global Innovation Index (GII) captures country’s innovation performance. The score is based on several measures, including knowledge creation, online creativity, available education and technology infrastructure [38].

## Results

### Substantive sex differences in occupational aspirations

**Fig 1:**
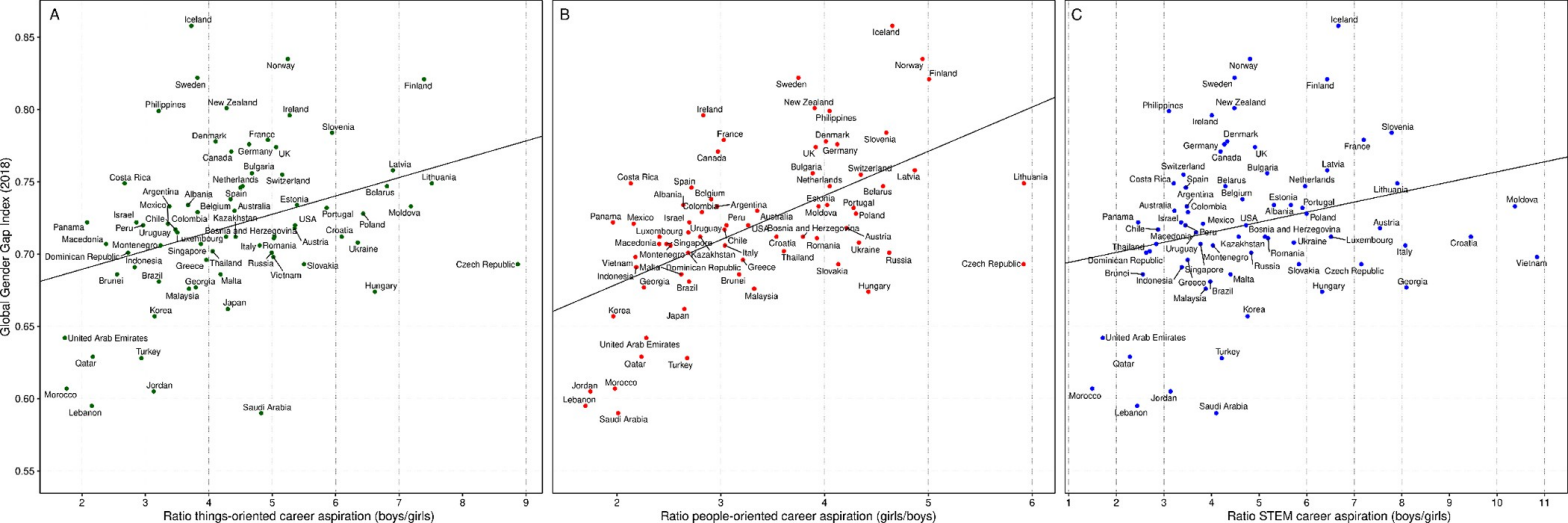
Ratios of boys to girls aspiring to work in things-oriented (green), people-oriented (red), and STEM occupations (blue). A: Each point represents the percentage of boys aspiring to work in a things-oriented occupation divided by the percentage of girls with the same aspiration and the country’s GGGI score. B: Like A, but now for the percentage of girls aspiring to work in a people-oriented occupation divided by the percentage of boys with the same aspiration. C: Like A, but now for non-organic STEM occupations only.

Confirming the first hypothesis, in all countries the percentage of boys aspiring to a things-oriented occupation was higher than the percentage of girls (Fig 1A). The gap was the smallest in the United Arab Emirates (UAE; boys 38.6%; girls 22.3%) and largest in Czechia (boys 56.2%; girls 6.3%).

Across countries, the median percentages of boys and girls aspiring to a things-oriented occupation were 37.4% and 8.7%, respectively (i.e., a median ratio of 4.3 boys for each girl). Confirming the second hypothesis, the ratio of boys to girls aspiring to a things-oriented occupation increased with increases in economic, political, and cultural opportunities for girls and women (i.e., women’s empowerment, as measured by the GGGI), *r*(69) = .348, *p* = .003 (Fig 1A).

Similarly, in all countries more adolescent girls than boys aspired to people-oriented occupations (Fig 1B). This ratio was smallest in Lebanon (boys 32.0%; girls 54.3%) and largest in Lithuania (7.1% boys; 42.1% girls). Across nations, the median percentages of girls and boys choosing a people-oriented occupation were 46.8% and 15.3%, respectively (i.e., a ratio of 3.0 girls for each boy). Again, the ratio increased with increases in women’s empowerment, *r*(69) = .563, *p* < .001 (Fig 1B).

A similar sex-specific pattern was found for STEM career aspirations, although these aspirations were less common than things-oriented aspirations. The international median percentage of students with a STEM career aspiration (boys and girls) was 10.1% compared to 23.4% with a things-oriented career aspiration.

STEM career aspirations were uncommon for girls (even in the more technically developed OECD nations, 2.9% of girls compared to 14.8% of boys). Girls’ low interest levels were also evident in countries that lead the world in the development of advanced technologies (Cornell University, INSEAD, and WIPO, 2018), such as Germany (2.3%, [1.5–3.0]), South Korea (2.9%, [2.3–3.6]), Great Britain (3.6%, [2.9–4.3]), and the US (3.8%, [3.0–4.6]). The lowest gender ratio in STEM career aspirations was found in Morocco (1.49, [1.3–1.7], 17.2% of boys, 11.5% of girls) and the largest in Vietnam (10.8, [6.6–15.1], 13.2% of boys, 1.2% of girls). Like things-oriented occupations, the STEM-gap ratios were often larger in countries with higher levels of women’s empowerment, as revealed by a positive correlation between the STEM gender ratio and GGGI, *r*(68) = .252, *p* = .035 (Fig 1C).

For clarity, based on the above, we also combined the more typical occupations aspired to by boys and girls into the sex-typical and sex-atypical categories. We define sex-typical occupational aspirations as boys aspiring to things-oriented or STEM careers and girls to people-oriented careers. Sex-atypical occupational aspirations are the opposite. Using these definitions, the percentage of students aspiring to sex-typical occupations varied from 26%[23.7%-28.0%] in Indonesia to 57%[55.7%-58.4%] in Slovenia (international median of 43%). Students who do not aspire to a sex-typical occupation do not necessarily aspire to a sex-atypical one, but may instead choose an occupation that is neither things, people, or STEM oriented. Hence, we also report the percentages of students with sex-atypical occupations. As expected, the percentages of students aspiring to sex-typical occupations were higher in countries with higher levels of women’s empowerment, *r*(68) = .350, *p* = .003. The percentages of students aspiring to sex-atypical occupations ranged from 7%[6.2%-7.8%] in Lithuania to 23%[21.2%-24.3%] in Lebanon (international median of 12%). This variation correlated negatively with women’s empowerment, *r*(68) = -.500, *p* < .001.

Across nations, as the percentage of students aspiring to a things-oriented occupation decreased, the percentage of students interested in non-things or non-people-oriented occupations (i.e., other) increased, but more so for boys (*r*[78] = -.807, *p* < .001) than for girls, *r*(78) = -.397, *p* < .001. Similarly, for girls but not for boys, as the percentage of girls aspiring to a people-oriented career decreased, the percentage aspiring to a non-people- or non-things-oriented career increased (*r* = -.900, *p* < .001). These patterns suggest greater flexibility in boys’ relative to girls’ things-oriented and girls’ relative to boys’ people-oriented occupational aspirations.

**Fig 2:**
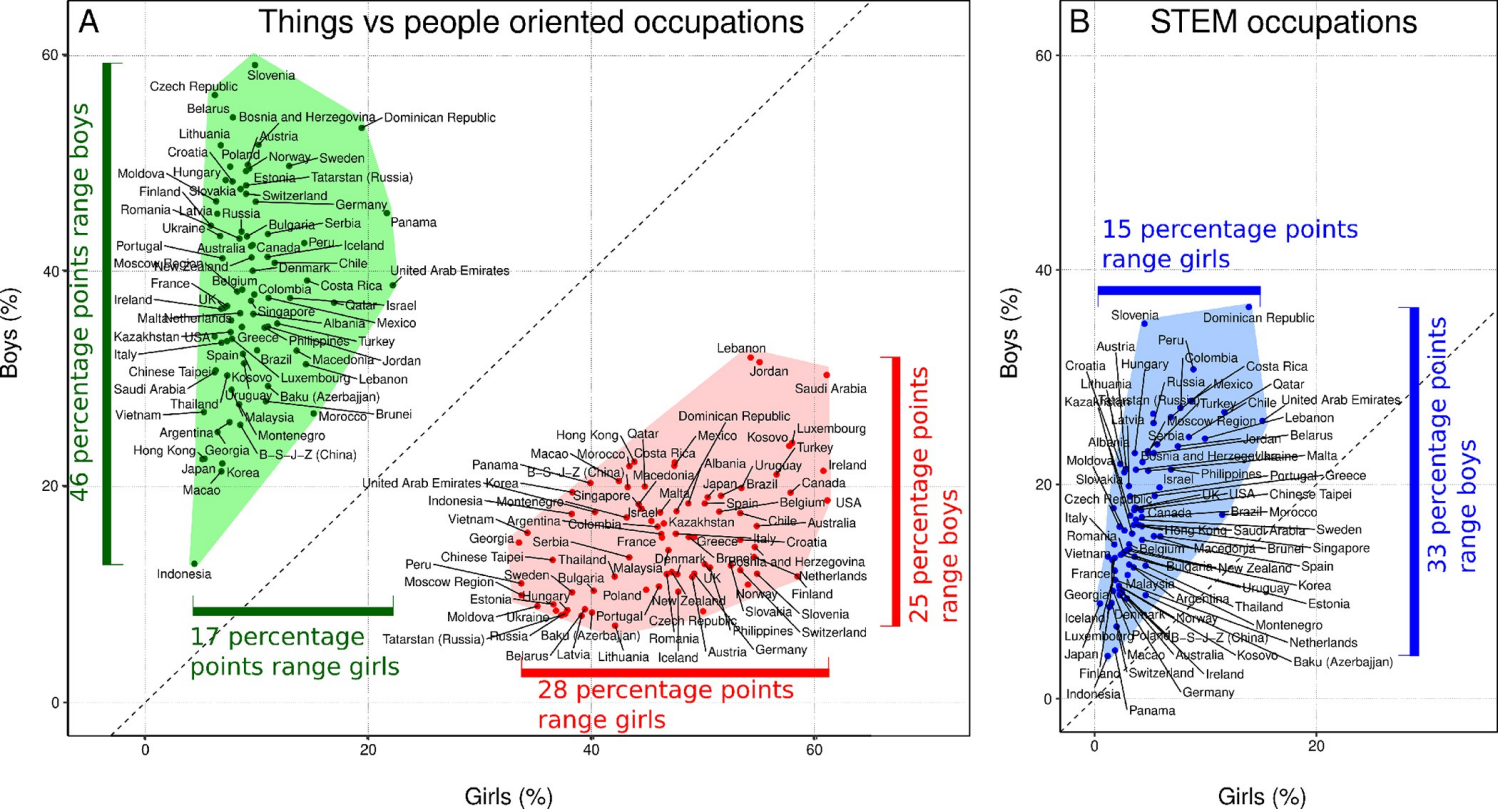
Percentage girls and boys aspiring to work in people-oriented occupations (panel A, red), things-oriented occupations (panel A, green) and STEM occupations (panel B, blue). Note that in all countries, more girls than boys aspire to a people-oriented occupation, hence all (red) points are below the line of equality (45°); similarly, in all countries, more boys than girls aspire to a things-oriented or STEM occupation, hence all green and blue points are above the lines of equality. Note that the international variation in things-oriented and STEM occupations is much larger for boys than for girls.

Indeed, there was greater cross-country variation in girls’ aspirations for working in people-oriented (ranging between 33% and 61%, *SD* = 7.1) than in things-oriented occupations, ranging between 4% and 22%, *SD* = 3.4, Levene’s Test, *F*(1,158) = 40.8, *p* < .001.Similarly, there was greater cross-country variation in boys’ aspirations to work in things-oriented (ranging between 13% and 59%, *SD* = 9.1) than people-oriented occupations, ranging between 7% and 32%, *SD* = 5.4, Levene’s Test, *F*(1,158) = 18.1, *p* < .001 (Fig 2). A similar difference in variability across countries was found for STEM-oriented occupations (*SD* of percentage of boys and girls with STEM career aspiration was 6.6 and 2.8, respectively).

The cross-country variation in occupational aspirations was related to two additional factors. First, the relation between GGGI and aspirations for a things-oriented occupation differed for boys and girls. More boys aspired to a things-oriented career in countries with a higher GGGI, *r*(69) = .418, *p* < .001, but this was not the case for girls, *r*(69) = −0.175, *ns*. Similarly, fewer boys aspired to a people-oriented career in countries with a higher GGGI, *r*(69) = −.563, *p* < .001, while this was not the case for girls, *r*(69) = .059, *ns*. Thus, the relation between the sex differences in things/people-related occupational aspirations and GGGI was influenced primarily by variation in boys’ aspirations. For the smaller subset of STEM occupations, the situation was as follows. Fewer students aspired to a STEM occupation in countries with a higher GGGI, *r*(68) = −.33, *p* = .005, but this effect was less pronounced for the relation between GGGI and the percentage of boys, *r*(68) = −.25, *p* = .035, than for the relation between GGGI and percentage of girls aspiring to a STEM career, *r*(68) = −0,44, *p* < .001. Especially for STEM, fewer boys and girls aspire to a STEM career in the more innovative countries (Global Innovation Index), *r*(68) = −.41, *p* < .001.

As noted in the Introduction, national wealth levels have been hypothesized as contributing to the sex differences in the things gap and women’s political and economic empowerment. The correlation between the sex-ratios in things-oriented occupations and PISA’s wealth index confirmed this, *r*(78) = .317, *p* = .004. The same is true for the correlation between the sex-ratios in people-oriented occupations and wealth, *r*(78) = .314, *p* = .005. In other words, increases in family wealth are associated with more sex-typical occupational aspirations.

The relation between boys’ aspirations and GGGI was also related to whether the occupations were blue collar or white collar. In countries with a higher GGGI, the percentage of boys (but not girls, due to outliers, see below) aspiring to things-oriented blue-collar jobs increased, *r*(69) = .524, *p* < .001. For boys, things-oriented blue-collar occupations ranged from 0.2% in Saudi Arabia to 33% in Czechia. For girls, the situation was different. In most countries with GGGI data available (*N = 54*), fewer than 1% of girls aspired to these occupations (ranging from 0% in Saudi Arabia to nearly 3% of girls in Sweden, Norway, and Vietnam). The correlation between GGGI and girls aspiring to things-oriented blue-collar occupations (*r* = .377, *p* = .001) was driven by two small groups of outlier countries, *N =* 4. Removing these data on both sides would render this correlation non-significant, *r(*65) = .199, *ns* (Norway, Sweden on one side and Saudi Arabia and Qatar on the other side).

### Parental occupational patterns

For each country, we analyzed sex differences in paternal and maternal occupations. The larger the ratio of girls to boys with people-oriented career aspirations, the larger the ratio of mothers to fathers in actual people-oriented occupations *r*(77) = .623, *p* < .001. In keeping with the results for students, the parental gender ratio in people-oriented occupations was larger in countries with a higher-levels of women’s empowerment, *r*(68) = .404, *p* < .001.

The pattern in things-oriented occupations was, however, the opposite of that seen in students. That is, the smaller the ratio of boys to girls in things-oriented career aspirations, the larger the ratio of fathers to mothers (median of 6.7 men for every woman) in actual things-oriented occupations *r*(78) = −.367, *p* < .001. This unexpected and interesting phenomenon shows, for example, that in most countries, the sex ratio of adult women’s (mothers) and men’s (fathers) actual occupations was much larger than seen in the occupational aspirations of students (see Discussion).

**Fig 3:**
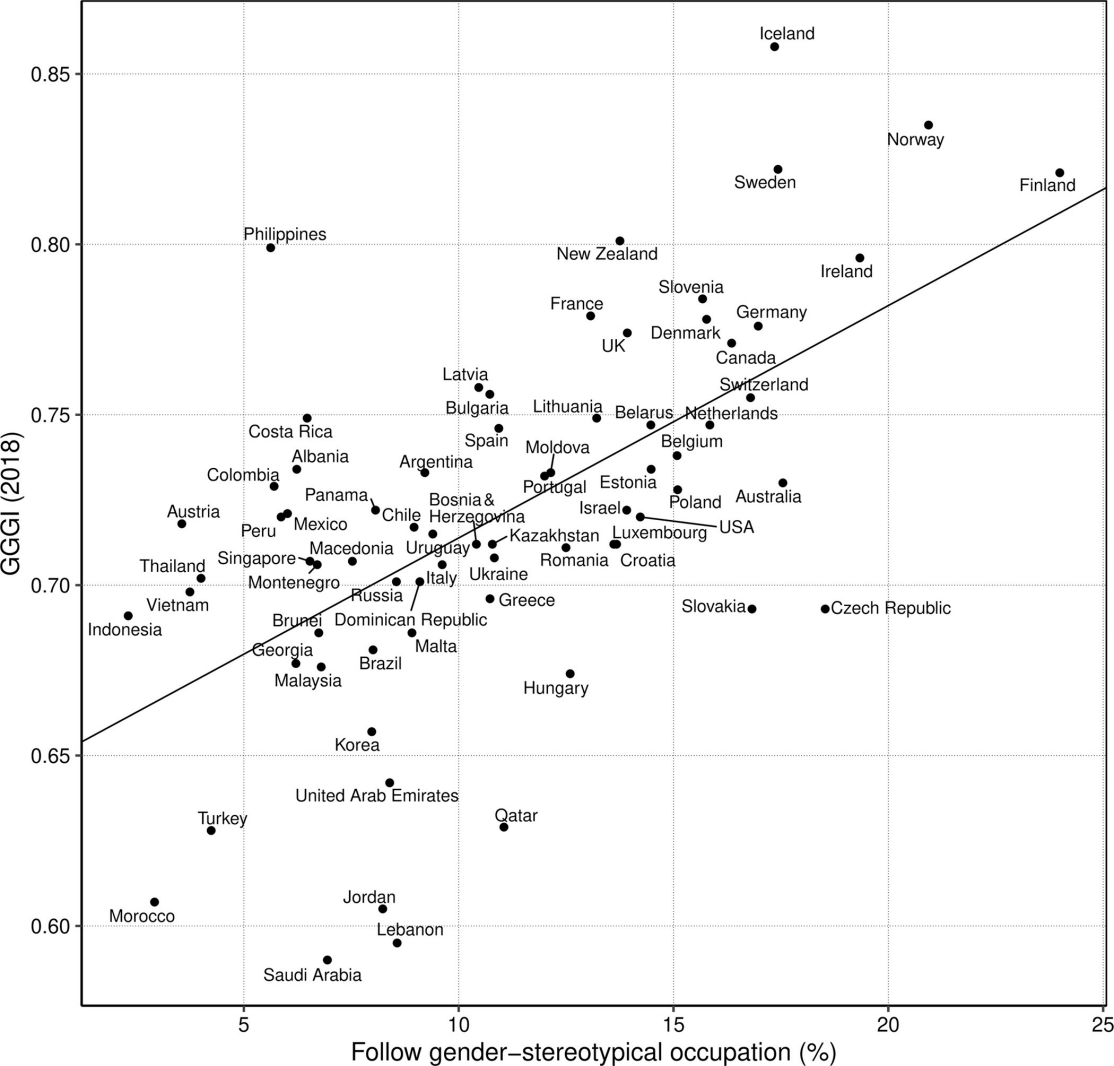
Percentage of students aspiring to a gender-stereotypical occupational category based on the occupation of the same-sex parent correlates with women’s empowerment (GGGI). For instance, in Finland 24% of students aspired to a gender-stereotypical occupation (boys aspiring to things-oriented or STEM occupations, and girls to people-oriented occupations) and their same-sex parent was employed in a similar occupational category.

Finally, we calculated for each country the percentage of students who aspired to an occupational category that was sex-typical and in the same occupational category as the same-sex parent (e.g., a boy aspiring to a things-oriented or STEM career with a father employed in a things-oriented or STEM career; or a girl aspiring to a people-oriented career with a mother employed in a people-oriented career). The results showed that students were more likely to express sex-typical occupational aspirations that were similar to their parents’ sex-typical occupations in countries with greater levels of women’s empowerment (GGGI), *r*(68) = .596, *p* < .001 (Fig 3) and in countries with greater levels of family wealth, *r*(77) = .689, *p* < .001.

### Mediation analysis

We tested the hypothesis (hypothesis 3) that wealth is an intermediate variable that explains the counter-intuitive relation between women’s empowerment and the percentage of students aspiring to sex-typical occupations. We carried out a mediation analysis using a bootstrapping procedure with 5,000 iterations (95% CI provided is based on the different samples created). The direct path effect was statistically significant, 0.2212, 95% CI [0.16,0.61], *p* = .031. The indirect effect was statistically significant, 0.1291, 95% CI [0.02, 0.29], *p* = .008. The mediation was partial with the proportion mediated 0.3685, 95% CI [0.08,0.90], *p* = .007.

#### Promotion of gender equality, high academic standards, and smaller sex differences in occupational aspirations

**Fig 4:**
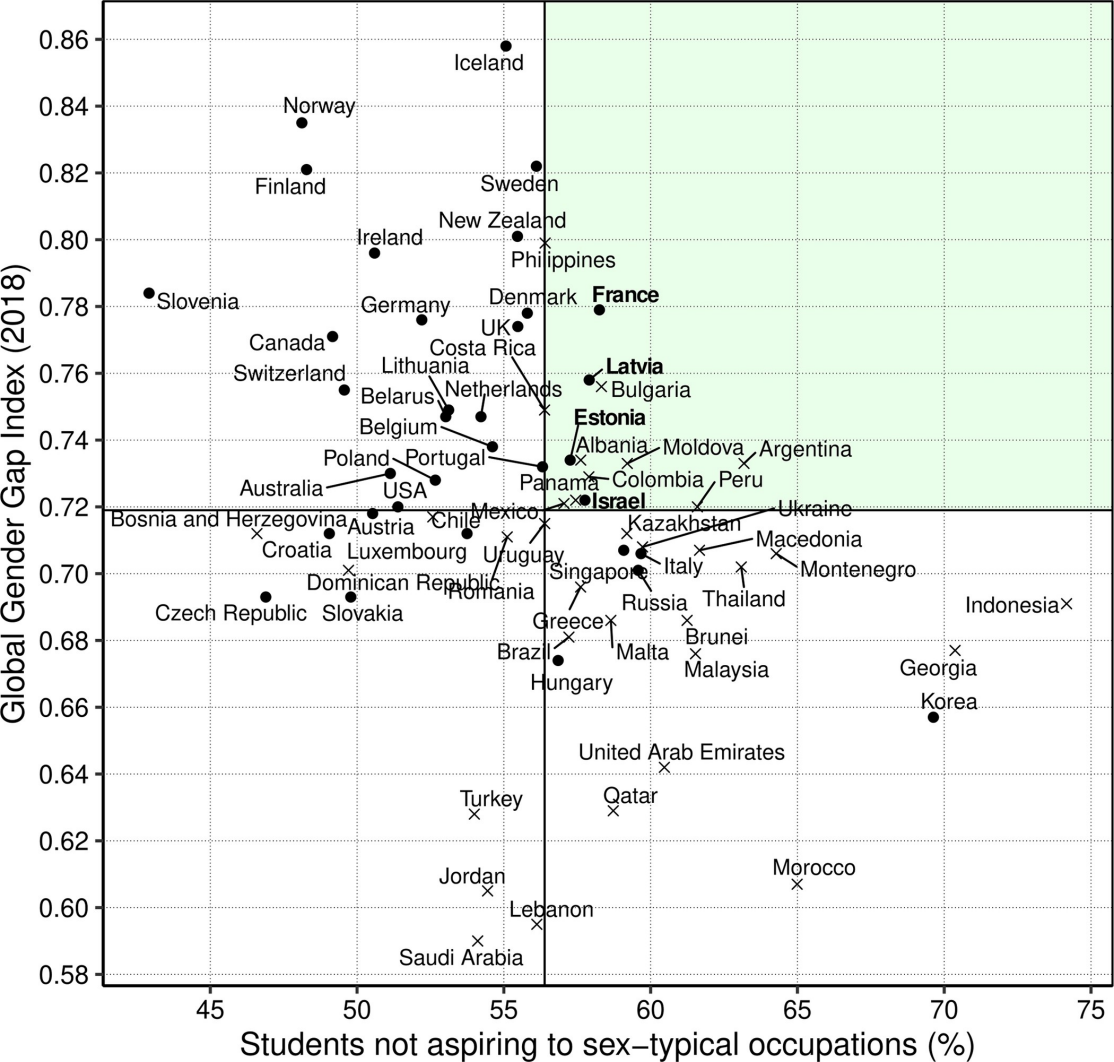
Countries that stand out in increasing the percentage of students not aspiring to sex-typical occupations with above average levels of women’s empowerment and educational achievement. Note that four (of 69) countries met these criteria, namely France, Latvia, Estonia, and Israel. The other countries in the green area had below average levels of educational achievement.

We sought to identify the factors that differentiated countries in terms of the percentage of students that aspired to a career that is not sex-typical while also considering the degree to which countries promoted women’s empowerment and relatively high academic standards. Because of the gender-equality paradox, there are arguably no straightforward ways to identify countries that stand out in terms of scoring high on all potentially important economic and social variables (e.g., women’s empowerment) and with high-achieving students. One approach is to identify groups of countries where many students are not aspiring to sex-typical occupations, have a relatively high level of girls and women’s empowerment, and where students’ academic achievement is above the international median. We did this by splitting the data into countries below and above the median values. The median values were 0.719 for women’s empowerment (GGGI), 472 for average PISA scores (across reading, mathematics, and achievement), and 56.4% for the percentage of students not aspiring to a sex-typical occupation. Estonia, Latvia, France, and Israel scored higher than the median on each of these three variables (Fig 4). In the Discussion, we elaborate on why these countries might have scored higher.

## Discussion

This large-scale and world-wide assessment revealed that adolescents’ occupational aspirations track previously reported adults’ sex-specific career interests and patterns [4]. In all 80 countries and economic regions included here, adolescent girls were more likely to aspire to a people-oriented than a things-oriented occupation (and vice versa for boys), a general pattern that is remarkably consistent with that found by Miner [10] a century ago. Further, we found that this sex difference is larger in countries with greater empowerment of girls and women, consistent with a so-called gender-equality paradox, which was previously found in university graduation rates and career choices [3, 6]. In other words, these differences appear well before adulthood.

We found that the latter correlations are mostly due to an increase in boys’ aspirations to enter things-oriented blue-collar careers and a decrease in boys’ aspirations to enter people-oriented careers in countries with greater women’s empowerment. In contrast, the percentages of girls aspiring to things-oriented jobs or people-oriented jobs did not systematically vary with national-levels of girls’ and women’s empowerment.

We combined the data from boys’ things-oriented or STEM-oriented occupational aspirations and girls’ people-oriented occupational aspirations into one sex-typical measure of aspirations and showed that sex-typical aspirations were more common in countries with greater levels of women’s empowerment. A mediation model showed that the counter-intuitive relation between women’s empowerment and sex-typical aspirations was partially explained by national levels of wealth, in keeping with our third hypothesis. Our interpretation of this result is that increased levels of women’s empowerment increase national wealth. The increased level of national wealth contributes to a situation in which students can aspire to careers that fit their interests rather than being largely based on economic security. We call this the Counter Intuitive Gender Empowerment Model (CIGEM).

We also found that the percentage of students aspiring to sex-typical occupations tracked the percentage of same-sex parents working in sex-typical occupations and both percentages increased with increases in women’s empowerment. The pattern suggests women’s gains in educational, political, and occupational spheres do not result in cross-generational increases in engagement in sex-atypical occupations (e.g., girls becoming electricians), and in fact just the opposite (cf., [38, 39]). At the same time, the sex ratio among adolescents aspiring to things-oriented occupations is often smaller than the ratio in parental occupations, which we elaborate on below. We will also discuss the theoretical and practical implications of our main findings as well as nuances in these and related findings; this discussion is particularly relevant within the context of sociopolitical targets to increase the number of students aspiring to non-stereotypical occupations, especially increasing girls’ and women’s participation in technical occupations.

### International variation

Across nations, there was a clear segregation of adolescent boys and girls in their relative aspiration to things-oriented (around four boys for each girl) as contrasted with people-oriented (around three girls to each boy) occupations. This was also the case for STEM career aspirations. The magnitude of these sex differences varied across countries and confirmed a gender-equality paradox [3, 6]; that is, the sex differences were often larger in countries in which women and girls have more opportunities in economic, educational, and political domains.

We add nuance to this paradox by showing that other factors are also related to the magnitude of these sex differences, especially to things-oriented occupational aspirations. In countries in which women’s economic and political empowerment is higher (i.e., high GGGI scores), the numbers of boys aspiring to skilled blue-collar occupations (e.g., electrician) is higher as well. The increased interest in these blue-collar occupations might in part be related to the higher level of economic and social development in these countries [39, 40]; that is, these occupations are often better paid and safer than they are in less developed ones [40, 41], especially for occupations that are traditionally dominated by men [41, 42]. For instance, Wu and colleagues [41, 42] found that in 2016 the occupational death rate (largely men) in the UAE (GGGI = 0.642) was 57 times higher than that found in Denmark (GGGI = 0.778).

### Sociocultural changes over time

There are several methodologically solid studies on occupational interests dating before the second world war [2–5, 11–15]. These studies provide an important backdrop against which the current results can be interpreted, especially given the substantive social and economic changes that accelerated after the second world war [42, 43]. Whatever the underlying causes of these changes, the contrast reveals two important patterns. First, it has been four generations since Miner’s [10] assessment of adolescents’ occupational interests and core sex differences have not changed much, despite dramatic social and economic changes since that time. Boys continue to express a greater interest in blue-collar and white-collar things-oriented occupations than do girls, and girls continue to show a greater interest in people-oriented occupations than do boys.

Second, a century ago, many girls expressed an interest in biology in school [2], but, except for nursing [43, 44], this did not translate into aspirations for careers in the life sciences, such as biologist or physician. In 1930, for instance, there were nearly 22 male physicians for every female physician, but one male nurse for every 50 female nurses [43, 44]. In the ensuing decades there were substantive improvements in women’s educational and occupational opportunities, especially in countries with high levels of women’s empowerment, that in turn broadened their aspirations.

The sociocultural changes over the past century appear to have broadened girls’ occupational and economic aspirations but, at the same time, these secular shifts are rarely toward male-typical things-oriented occupations. Although there are countries where hardly any boys or girls aspire to blue-collar things-oriented occupations (e.g., Saudi Arabia or Qatar), there are highly developed nations where a quarter of boys aspire to such occupations (e.g., Finland and Norway); at the same time, only 3% of girls aspire to a blue-collar things-oriented occupation in the country with the highest percentage of girls with such aspirations (Vietnam).

The consistency of these core sex differences across generations [10], changes in labor market participation [3], and the cross-national consistency found here and elsewhere [4, 6] suggests that, apart from obvious socioeconomic influences, there is a biological contribution to these occupational interests. In some contexts, risk of injury [41, 42] or low status [44, 45] will reduce the attractiveness of some blue-collar occupations, but when the risks are lower and the rewards are higher these occupations become attractive, but almost exclusively to males. Indeed, broad occupational interests appear to correlate with prenatal exposure to androgens [18]. This does not necessarily mean that the sex differences in interests in specific occupations are biologically influenced but rather sex differences in occupation-relevant abilities (e.g., mechanical reasoning or spatial ability [46, 47]) or broader factors, such as sensitivity to people or the physical properties of objects, might lead to differences in interest in the occupations available in any social or historical context [44, 45, 47, 48].

It should be pointed out, however, that the sex ratio among students with a career aspiration for a things-oriented occupation is often smaller than the actual occupational patterns observed among the parents of these children. This can be interpreted in different ways. For example, one possibility is that once women complete their preparation for a things-oriented occupation, barriers (e.g., in hiring) keep or drive them out of these occupations. Alternatively, it is possible that many women studying for things-oriented occupations use their knowledge and skills in the educational sector (e.g., teaching physics), which would then count as a people-oriented occupation. It is also possible that women who train for and enter these occupations do not stay in them (c.f., [48, 49]). Finally, it is possible that there have been recent changes in students’ aspirations that explain these generational differences. Unfortunately, we did not have the data needed to differentiate among these explanations and thus these questions will need to be addressed in follow-up studies.

### Sociopolitical implications

Policy makers have regularly expressed a desire to reduce the number of students choosing stereotypical careers (e.g., [50]) or to increase the number of girls aspiring to and women entering technical occupations, especially STEM occupations [22]. The results of this study and related ones reveal a policy-relevant conundrum [3, 4, 6, 50]. Generally speaking, more developed and gender equal nations are better than less developed nations in attracting boys to more established things-oriented (often blue-collar) occupations, but they fail to attract girls to these areas. This problem is also occurring for the subset of things-oriented STEM occupations. In fact, the problem for STEM is even more profound, given that we found that STEM aspirations decline for both boys and girls in more developed, innovative, and gender-equal nations.

This means that the more developed and gender-equal countries are a long way from closing the much-discussed gender gap in STEM and in many other relatively segregated occupations [51]. As noted, women have a particularly low engagement with things-oriented and certain STEM fields, confirming earlier studies [3, 6]. The current results extend this pattern to younger students who are still at an age where they can choose a technical career, whether STEM or applied blue collar.

Unlike previous studies, this study offers specific information on which factors might provide the best options for interventions, should that be the goal. First, our finding of a clear gender segregation in the occupational aspirations of 15- and 16-year-olds indicates that any targeted information about things-oriented and STEM occupations needs to be provided at an earlier age, although there is no guarantee that these interventions would be effective. Currently, there are many STEM initiatives for adolescent girls (e.g., STEM day initiatives in many European countries, [52, 53]), but these might be too late to generate interest in these areas.

Second, much of the international variation in the aspirations gap is related to an increasing number of boys aspiring to a thing-oriented blue-collar job when the socioeconomic climate affords this − the most likely reason this correlates with women’s empowerment (i.e., GGGI) is that countries that afford better working conditions for women, including issues such as worker protections, also afford better working conditions in predominantly male blue-collar industries (more reliable jobs and health and safety [41, 42]).

Third, although we suggest that biological factors likely contribute to the strong sex differences in career aspirations, this does not mean that the magnitude of the currently observed sex differences is inevitable. If correct, it means that these sex differences are more difficult to counter than when the causes were purely social in nature (as is often assumed, for a review, see [19]). While the assumption that the causes are social in nature seems to be easier to communicate and digest in modern society, the assumption can lead to a weaker approach in dealing with gender segregation than would be required if biology does play a role. For instance, if there are substantive biological influences on interest in blue-collar things-oriented occupations, then interventions to encourage girls to enter these occupations might not be cost effective. At the same time, many girls do enter high-paying white-collar occupations that are neither things- nor people-oriented (e.g., management). Interventions that better prepare girls for such occupations and the associated long-term gains in income might be more fruitful, should interventions be deemed appropriate.

The latter point raises the question of why such interventions are deemed appropriate in the first place. The scope of this question is too broad to discuss here, but we would like to note that the potential benefits of gender parity in occupations are not necessarily the same for individuals as for organizations/countries. Individual students in wealthy nations with strong social safety nets might choose more in accordance with biologically influenced occupational interests (along the people things dimension) than in accordance with political ideals–this explains the relatively low engagement in sex-atypical occupations in these nations, despite no lack of awareness of the ideals of gender parity in all sectors of society.

Relatedly, we proposed a method to identify potential model countries that not only have a relatively large number of students not choosing a sex-typical occupation, but also have relatively high educational achievement and overall women’s empowerment. Tallying the percentage of students not choosing sex-typical occupations (although these are not necessarily sex-atypical occupations) is more inclusive than merely focusing on women entering STEM occupations, as is commonly done. Our approach includes a wider spectrum of occupations and skill levels and not only focuses on girls, but also on boys not aspiring to sex-typical occupations.

We identified Estonia, Latvia, France, and Israel as potential model countries in this regard. We can only speculate about what these countries have in common, but we would rather note that these countries are diverse in terms of size and sociocultural context. Future research needs to determine if there are specific sociocultural, educational, or economic factors that contribute to the relatively high percentage of students aspiring to enter sex-atypical occupations.

## Supporting information

S1 Table Detailed country data. For each country, we list the number of boys and girls with career aspiration data, the percentages of boys and girls with a things, people, or STEM oriented career aspiration (as well as the ratios), the overall PISA score (average of mathematics, science, and reading), the Global Gender Gap Index (GGGI), and the national wealth level.
(DOCX)
