# Supplementary material for: Sex differences in adolescents’ occupational aspirations: Variations across time and place
Source: PLoS One. 2022 Jan 26;17(1):e0261438. doi: 10.1371/journal.pone.0261438 (PMC8791526; doi:10.1371/journal.pone.0261438)
Supplement: S1 Table — For each country, we list the number of boys and girls with career aspiration data, the percentages of boys and girls with a things, people, or STEM oriented career aspiration (as well as the ratios), the overall PISA score (average of mathematics, science, and reading), the Global Gender Gap Index (GGGI), and the national wealth level. (DOCX) [file pone.0261438.s001.docx]

| *Country* | *Participants* | | *Things-oriented* | | | *People-oriented* | | | *STEM* | | | *PISA* | *GGGI* | *Wealth* |
| --- | --- | --- | --- | --- | --- | --- | --- | --- | --- | --- | --- | --- | --- | --- |
|  | *boysn* | *girls*  *n* | *boys %* | *girls %* | *ratio* | *girls %* | *boys %* | *ratio* | *boys %* | *girls %* | *ratio* |  |  |  |
| Albania | 3167 | 3192 | 35.9 | 9.8 | 3.7 | 48.7 | 18.5 | 2.6 | 17.1 | 3.2 | 5.3 | 423 | 0.734 | -1.26 |
| Argentina | 6232 | 5743 | 25.9 | 7.7 | 3.4 | 46.3 | 15.6 | 3 | 12.2 | 3.5 | 3.5 | 401 | 0.733 | -1.08 |
| Australia | 7075 | 7198 | 42.2 | 9.6 | 4.4 | 54.8 | 16.4 | 3.4 | 9.4 | 2.9 | 3.2 | 502 | 0.73 | 0.66 |
| Austria | 3321 | 3481 | 49.8 | 9.3 | 5.4 | 49 | 11.6 | 4.2 | 21.4 | 2.8 | 7.5 | 491 | 0.718 | 0.12 |
| B-S-J-Z (China) | 5775 | 6283 | 25.6 | 8.6 | 3 | 43.3 | 20 | 2.2 | 10.2 | 2.3 | 4.5 | 584 | - | -0.82 |
| Baku (Azerbajjan) | 3262 | 3565 | 29.2 | 11.1 | 2.6 | 40 | 8.3 | 4.8 | 9.7 | 4.6 | 2.1 | 413 | - | -1.17 |
| Belarus | 2772 | 3031 | 54.2 | 8 | 6.8 | 39.4 | 8.6 | 4.6 | 22.9 | 5.3 | 4.3 | 473 | 0.747 | -0.80 |
| Belgium | 4271 | 4204 | 38.2 | 8.8 | 4.3 | 51.5 | 17.7 | 2.9 | 14.4 | 3.1 | 4.6 | 494 | 0.738 | 0.16 |
| Bosnia and Herzegovina | 3148 | 3332 | 51.6 | 10.3 | 5 | 54.7 | 14.4 | 3.8 | 22.1 | 4.3 | 5.1 | 406 | 0.712 | -0.63 |
| Brazil | 5478 | 5213 | 32.5 | 10.1 | 3.2 | 51.6 | 19.2 | 2.7 | 16.9 | 4.3 | 4 | 409 | 0.681 | -1.37 |
| Brunei | 3383 | 3445 | 27.8 | 10.9 | 2.6 | 48.7 | 15.3 | 3.2 | 15.1 | 5.9 | 2.6 | 428 | 0.686 | -0.22 |
| Bulgaria | 2533 | 2761 | 43.1 | 9.2 | 4.7 | 40.2 | 10.3 | 3.9 | 13.7 | 2.6 | 5.2 | 440 | 0.756 | -0.36 |
| Canada | 11307 | 11344 | 42.3 | 9.7 | 4.4 | 57.9 | 19.5 | 3 | 17.6 | 4.2 | 4.2 | 524 | 0.771 | 0.46 |
| Chile | 3814 | 3807 | 40.7 | 11.7 | 3.5 | 53.3 | 17.6 | 3 | 24.4 | 8.5 | 2.9 | 441 | 0.717 | -0.79 |
| Chinese Taipei | 3624 | 3619 | 30.7 | 6.4 | 4.8 | 36.5 | 13.2 | 2.8 | 17.6 | 3.6 | 4.9 | 522 | - | -0.50 |
| Colombia | 3857 | 3665 | 37.7 | 9.9 | 3.8 | 45.9 | 16.3 | 2.8 | 27.1 | 7.7 | 3.5 | 410 | 0.729 | -1.67 |
| Costa Rica | 3618 | 3603 | 39.0 | 14.6 | 2.7 | 47.5 | 22.3 | 2.1 | 27.8 | 8.7 | 3.2 | 417 | 0.749 | -1.13 |
| Croatia | 3311 | 3298 | 48.2 | 7.9 | 6.1 | 53.4 | 15.1 | 3.5 | 21.9 | 2.3 | 9.5 | 473 | 0.712 | -0.42 |
| Czech Republic | 3518 | 3501 | 56.2 | 6.3 | 8.9 | 50 | 8.5 | 5.9 | 16 | 2.2 | 7.1 | 492 | 0.693 | -0.13 |
| Denmark | 3816 | 3841 | 39.9 | 9.7 | 4.1 | 47.7 | 11.9 | 4 | 9.7 | 2.2 | 4.3 | 507 | 0.778 | 0.98 |
| Dominican Republic | 2890 | 2784 | 53.2 | 19.5 | 2.7 | 47.6 | 17.7 | 2.7 | 36.5 | 13.9 | 2.6 | 350 | 0.701 | -1.70 |
| Estonia | 2651 | 2665 | 49.2 | 9.1 | 5.4 | 36.6 | 9.1 | 4 | 13.4 | 2.4 | 5.7 | 528 | 0.734 | -0.10 |
| Finland | 2772 | 2877 | 44.1 | 6 | 7.4 | 58.5 | 11.7 | 5 | 8.6 | 1.3 | 6.4 | 522 | 0.821 | 0.20 |
| France | 3078 | 3230 | 36.7 | 7.4 | 4.9 | 46.3 | 15.3 | 3 | 13.1 | 1.8 | 7.2 | 494 | 0.779 | -0.05 |
| Georgia | 2682 | 2890 | 25.0 | 6.6 | 3.8 | 33.5 | 14.8 | 2.3 | 12.9 | 1.6 | 8.1 | 395 | 0.677 | -1.07 |
| Germany | 2525 | 2926 | 46.3 | 10 | 4.6 | 49.2 | 11.9 | 4.1 | 9.7 | 2.3 | 4.3 | 512 | 0.776 | 0.09 |
| Greece | 3178 | 3225 | 34.7 | 8.8 | 4 | 49.2 | 15.3 | 3.2 | 18.9 | 5.4 | 3.5 | 456 | 0.696 | -0.33 |
| Hong Kong | 2955 | 3082 | 22.5 | 5.4 | 4.2 | 43.9 | 22.3 | 2 | 16.2 | 3.5 | 4.6 | 539 | - | -0.46 |
| Hungary | 2605 | 2527 | 48.4 | 7.3 | 6.6 | 37.8 | 8.6 | 4.4 | 22.9 | 3.6 | 6.3 | 480 | 0.674 | -0.26 |
| Iceland | 1656 | 1640 | 41.2 | 11.1 | 3.7 | 47.8 | 10.3 | 4.7 | 12 | 1.8 | 6.7 | 488 | 0.858 | 0.43 |
| Indonesia | 6240 | 5858 | 12.7 | 4.5 | 2.8 | 38.2 | 17.5 | 2.2 | 4 | 1.2 | 3.4 | 384 | 0.691 | -2.32 |
| Ireland | 2777 | 2800 | 36.4 | 6.9 | 5.3 | 60.8 | 21.5 | 2.8 | 9.9 | 2.5 | 4 | 507 | 0.796 | 0.18 |
| Israel | 3544 | 3079 | 37.4 | 13.1 | 2.9 | 45.4 | 16.8 | 2.7 | 19.7 | 5.9 | 3.4 | 479 | 0.722 | 0.08 |
| Italy | 5680 | 6105 | 33.3 | 6.9 | 4.8 | 47.6 | 15.7 | 3 | 14.4 | 1.8 | 8.1 | 479 | 0.706 | -0.12 |
| Japan | 3120 | 2989 | 22.5 | 5.2 | 4.3 | 50.4 | 19 | 2.6 | 8.9 | 0.5 | 18.1 | 526 | 0.662 | -0.41 |
| Jordan | 4619 | 4344 | 34.8 | 11.1 | 3.1 | 55.1 | 31.6 | 1.7 | 23.5 | 7.5 | 3.1 | 425 | 0.605 | -1.03 |
| Kazakhstan | 9576 | 9931 | 34.3 | 7.7 | 4.4 | 46.5 | 16.6 | 2.8 | 15.4 | 3.4 | 4.6 | 405 | 0.712 | -1.11 |
| Korea | 3191 | 3459 | 22.1 | 7 | 3.1 | 38.3 | 19.5 | 2 | 13.8 | 2.9 | 4.8 | 522 | 0.657 | -0.45 |
| Kosovo | 2457 | 2601 | 32.2 | 8.9 | 3.6 | 57.7 | 23.8 | 2.4 | 10.5 | 2.2 | 4.8 | 367 | - | -0.90 |
| Latvia (LSS) | 2685 | 2618 | 45.2 | 6.6 | 6.9 | 39.1 | 8 | 4.9 | 19.8 | 3.1 | 6.4 | 490 | 0.758 | -0.35 |
| Lebanon | 3079 | 2535 | 31.3 | 14.5 | 2.2 | 54.3 | 32 | 1.7 | 24.3 | 9.9 | 2.4 | 395 | 0.595 | -0.72 |
| Lithuania | 3377 | 3508 | 51.6 | 6.9 | 7.5 | 42.1 | 7.1 | 5.9 | 21.1 | 2.7 | 7.9 | 483 | 0.749 | -0.30 |
| Luxembourg | 2594 | 2636 | 33.6 | 7.9 | 4.3 | 58 | 24.1 | 2.4 | 11.9 | 1.8 | 6.5 | 480 | 0.712 | 0.32 |
| Macao | 1862 | 1913 | 21.3 | 6.9 | 3.1 | 42.5 | 20.5 | 2.1 | 8.9 | 1.5 | 5.8 | 544 | - | -0.54 |
| Macedonia | 2596 | 2973 | 32.5 | 13.7 | 2.4 | 44.2 | 18.4 | 2.4 | 15.2 | 5.3 | 2.8 | 407 | 0.707 | -0.54 |
| Malaysia | 3131 | 2980 | 28.9 | 7.8 | 3.7 | 46.9 | 14.1 | 3.3 | 11.5 | 3 | 3.9 | 435 | 0.676 | -1.38 |
| Malta | 1612 | 1751 | 36.0 | 8.6 | 4.2 | 46.2 | 17.6 | 2.6 | 21.3 | 4.8 | 4.4 | 471 | 0.686 | 0.45 |
| Mexico | 3826 | 3473 | 37.4 | 11.1 | 3.4 | 47.4 | 22 | 2.2 | 26.3 | 6.9 | 3.8 | 423 | 0.721 | -1.48 |
| Moldova | 2621 | 2746 | 46.4 | 6.5 | 7.2 | 35.2 | 8.9 | 3.9 | 17.8 | 1.7 | 10.4 | 427 | 0.733 | -1.21 |
| Montenegro | 3240 | 3426 | 27.5 | 8.5 | 3.2 | 43.2 | 17.1 | 2.5 | 12.5 | 3.1 | 4 | 427 | 0.706 | -0.51 |
| Morocco | 3262 | 3552 | 26.7 | 15.2 | 1.8 | 43.4 | 21.9 | 2 | 17.2 | 11.5 | 1.5 | 377 | 0.607 | -1.88 |
| Moscow Region | 970 | 1046 | 38.0 | 8.3 | 4.6 | 33.7 | 9.9 | 3.4 | 22.9 | 4.8 | 4.8 | 491 | - | -0.29 |
| Netherlands | 2330 | 2435 | 35.3 | 7.8 | 4.5 | 54.6 | 13.5 | 4 | 11.1 | 1.9 | 6 | 516 | 0.747 | 0.43 |
| New Zealand | 3154 | 3019 | 41.2 | 9.6 | 4.3 | 47.2 | 12.1 | 3.9 | 14 | 3.1 | 4.5 | 503 | 0.801 | 0.42 |
| Norway | 2880 | 2933 | 49.4 | 9.4 | 5.2 | 54 | 10.9 | 4.9 | 10.5 | 2.2 | 4.8 | 501 | 0.835 | 0.67 |
| Panama | 3173 | 3097 | 45.3 | 21.7 | 2.1 | 39.9 | 20.3 | 2 | 4.5 | 1.8 | 2.5 | 379 | 0.722 | -1.64 |
| Peru | 3000 | 3086 | 42.5 | 14.3 | 3 | 33.7 | 11 | 3.1 | 30.7 | 8.9 | 3.5 | 416 | 0.72 | -2.04 |
| Philippines | 3868 | 3365 | 34.7 | 10.8 | 3.2 | 50.6 | 12.5 | 4 | 21.4 | 6.9 | 3.1 | 358 | 0.799 | -2.20 |
| Poland | 2857 | 2768 | 49.6 | 7.7 | 6.4 | 44.9 | 10.4 | 4.3 | 10 | 1.7 | 6 | 516 | 0.728 | -0.16 |
| Portugal | 2944 | 2988 | 41.1 | 7 | 5.9 | 46.1 | 10.8 | 4.3 | 18.9 | 3.2 | 5.9 | 492 | 0.732 | -0.09 |
| Qatar | 6954 | 6874 | 37.0 | 17 | 2.2 | 44.8 | 20 | 2.2 | 26.7 | 11.7 | 2.3 | 428 | 0.629 | 0.41 |
| Romania | 2444 | 2631 | 42.9 | 8.5 | 5 | 46.8 | 11.9 | 3.9 | 13.6 | 2.6 | 5.2 | 434 | 0.711 | -0.64 |
| Russian Federation | 3861 | 3747 | 43.6 | 8.7 | 5 | 37.3 | 8.1 | 4.6 | 25.7 | 5.3 | 4.8 | 483 | 0.701 | -0.55 |
| Saudi Arabia | 2992 | 3144 | 30.5 | 6.3 | 4.8 | 61.1 | 30.4 | 2 | 16.2 | 4 | 4.1 | 391 | 0.59 | -0.05 |
| Serbia | 3272 | 3337 | 43.3 | 11.1 | 3.9 | 43.4 | 13.5 | 3.2 | 23.1 | 4.8 | 4.8 | 449 | - | -0.62 |
| Singapore | 3277 | 3399 | 37.1 | 9.6 | 3.9 | 44.5 | 18 | 2.5 | 16.1 | 4.3 | 3.8 | 559 | 0.707 | 0.01 |
| Slovak Republic | 3002 | 2963 | 47.5 | 8.6 | 5.5 | 52.5 | 12.7 | 4.1 | 15.7 | 2.7 | 5.8 | 475 | 0.693 | -0.28 |
| Slovenia | 2993 | 3408 | 59.0 | 9.9 | 5.9 | 54.9 | 11.9 | 4.6 | 35 | 4.5 | 7.8 | 502 | 0.784 | -0.01 |
| Spain | 17956 | 17987 | 33.4 | 7.4 | 4.5 | 50.2 | 18.4 | 2.7 | 14.8 | 4.3 | 3.5 | - | 0.746 | 0.01 |
| Sweden | 2763 | 2741 | 49.7 | 13 | 3.8 | 38.3 | 10.2 | 3.7 | 16.7 | 3.7 | 4.5 | 508 | 0.822 | 0.43 |
| Switzerland | 2789 | 3033 | 47.1 | 9.1 | 5.2 | 53.4 | 12.3 | 4.3 | 6.7 | 2 | 3.4 | 499 | 0.755 | 0.14 |
| Tatarstan (Russia) | 2906 | 2910 | 47.9 | 9.1 | 5.2 | 37.6 | 8.2 | 4.6 | 26.6 | 5.3 | 5 | 470 | - | -0.56 |
| Thailand | 4693 | 3940 | 30.2 | 7.4 | 4.1 | 42.1 | 11.7 | 3.6 | 12.4 | 4.6 | 2.7 | 420 | 0.702 | -1.31 |
| Turkey | 3396 | 3494 | 35.0 | 11.9 | 2.9 | 56.6 | 21.1 | 2.7 | 23.7 | 5.6 | 4.2 | 465 | 0.628 | -1.34 |
| Ukraine | 2857 | 3141 | 43.2 | 6.8 | 6.3 | 36.8 | 8.5 | 4.3 | 21.4 | 3.7 | 5.7 | 466 | 0.708 | -0.89 |
| United Arab Emirates | 9380 | 9897 | 38.6 | 22.3 | 1.7 | 40.3 | 17.7 | 2.3 | 25.9 | 15.1 | 1.7 | 443 | 0.642 | 0.50 |
| United Kingdom | 6996 | 6822 | 36.5 | 7.2 | 5.1 | 50.2 | 12.8 | 3.9 | 17.8 | 3.6 | 4.9 | 506 | 0.774 | 0.42 |
| United States | 2376 | 2462 | 33.9 | 6.3 | 5.4 | 61.2 | 18.7 | 3.3 | 17.8 | 3.8 | 4.7 | 499 | 0.72 | 0.44 |
| Uruguay | 2732 | 2531 | 31.3 | 8.9 | 3.5 | 53.5 | 19.9 | 2.7 | 13.3 | 3.6 | 3.7 | 430 | 0.715 | -1.14 |
| Vietnam | 2780 | 2597 | 26.8 | 5.3 | 5 | 34.3 | 15.7 | 2.2 | 13.2 | 1.2 | 10.8 | - | 0.698 | -1.99 |
